# Probing the Dual Role of Ca^2+^ in the *Allochromatium tepidum* LH1–RC Complex by Constructing and Analyzing Ca^2+^-Bound and Ca^2+^-Free LH1 Complexes

**DOI:** 10.3390/biom15010124

**Published:** 2025-01-14

**Authors:** Mei-Juan Zou, Shuai Sun, Guang-Lei Wang, Yi-Hao Yan, Wei Ji, Zheng-Yu Wang-Otomo, Michael T. Madigan, Long-Jiang Yu

**Affiliations:** 1 Photosynthesis Research Center, Key Laboratory of Photobiology, Institute of Botany, Chinese Academy of Sciences, Beijing 100093, China; 2021101369@mails.cust.edu.cn (S.S.); wangguanglei20@mails.ucas.ac.cn (G.-L.W.); yanyihao18@mails.ucas.ac.cn (Y.-H.Y.); 2 School of Life Science and Technology, Changchun University of Science and Technology, Changchun 130022, China; jiweism@cust.edu.cn; 3 University of Chinese Academy of Sciences, Beijing 100049, China; 4 Faculty of Science, Ibaraki University, Mito 310-8512, Japan; wang@ml.ibaraki.ac.jp; 5 Department of Microbiology, School of Biological Sciences, Southern Illinois University, Carbondale, IL 62901, USA; madigan@siu.edu

**Keywords:** light-harvesting 1 (LH1) complex, purple bacterium, *Q_y_* absorption

## Abstract

The genome of the mildly thermophilic hot spring purple sulfur bacterium, *Allochromatium* (*Alc*.) *tepidum*, contains a multigene *pufBA* family that encodes a series of α- and β-polypeptides, collectively forming a heterogeneous light-harvesting 1 (LH1) complex. The *Alc. tepidum* LH1, therefore, offers a unique model for studying an intermediate phenotype between phototrophic thermophilic and mesophilic bacteria, particularly regarding their LH1 *Qy* transition and moderately enhanced thermal stability. Of the 16 α-polypeptides in the *Alc. tepidum* LH1, six α1 bind Ca^2+^ to connect with β1- or β3-polypeptides in specific Ca^2+^-binding sites. Here, we use the purple bacterium *Rhodospirillum rubrum* strain H2 as a host to express Ca^2+^-bound and Ca^2+^-free *Alc. tepidum* LH1-only complexes composed of α- and β-polypeptides that either contain or lack the calcium-binding motif WxxDxI; purified preparations of each complex were then used to test how Ca^2+^ affects their thermostability and spectral features. The cryo-EM structures of both complexes were closed circular rings consisting of 14 αβ-polypeptides. The *Q_y_* absorption maximum of Ca^2+^-bound LH1 (α1/β1 and α1/β3) was at 894 nm, while that of Ca^2+^-free (α2/β1) was at 888 nm, indicating that Ca^2+^ imparts a *Q_y_* transition of 6 nm. Crucially for the ecological success of *Alc. tepidum*, Ca^2+^-bound LH1 complexes were more thermostable than Ca^2+^-free complexes, indicating that calcium plays at least two major roles in photosynthesis by *Alc. tepidum*—improving photocomplex stability and modifying its spectrum.

## 1. Introduction

Phototrophic organisms have evolved diverse strategies for conducting photosynthesis since appearing on Earth at least three billion years ago [1]. In purple phototrophic bacteria, the molecular machinery of photosynthesis is typically composed of at least one and, in some cases, two photocomplexes. All species contain a light-harvesting 1−reaction center (LH1−RC) core complex, and many also contain a peripheral light-harvesting complex (LH2). Solar energy absorbed by the light-harvesting complexes is transmitted to the RC, where charge separation initiates electron transfer and eventually generates the proton motive force [2,3].

Recent advances in structural biology have provided many high-resolution three-dimensional structures of photocomplexes from purple bacteria, including the RC, LH1−RC, and LH2 complexes [4,5,6,7,8,9]. Because the LH1−RC complex is universally present in purple bacteria [3], its structure and function are key to understanding molecular mechanisms of photosynthesis. Comparisons of amino acid sequences in photocomplex polypeptides have revealed that while RCs are highly conserved, light-harvesting complexes exhibit substantial structural diversity. This diversity enables phototrophs to optimize the spectral and physical properties of their photocomplexes in order to photosynthesize in a variety of habitats, including many extreme environments [10,11].

LH1−RC complexes share a similar overall architecture, forming a closed or open ring surrounding the RC. The fundamental unit of the LH1−RC is the αβ-heterodimer consisting of transmembrane helices of an α- and β-polypeptide that bind bacteriochlorophyll (BChl) and carotenoids. Variability in the type and quantities of BChls and carotenoids within these units results in structural differences in the mature protein complexes [11]. Although the overall structure of the peripheral LH2 complex is similar to that of LH1, LH2 complexes form a substantially smaller ring, do not surround an RC, and exhibit two *Q_y_* absorption bands with maxima at ~800 and ~850 nm, respectively. By contrast, LH1 complexes containing BChl *a* or *b* show a single *Q_y_* absorption around ~880 nm and ~1000 nm, respectively [12,13].

In certain purple bacteria, LH1 displays significant changes in spectral properties and thermostability due to the binding of cofactors, such as calcium ions [7,9,14]. In the extensively studied purple sulfur bacterium *Thermochromatium* (*Tch.*) *tepidum*, its LH1 complex binds 16 calcium ions, which help maintain the close association of adjacent LH1 subunits, contributing to the stability of the complex [9]. These interactions redshift LH1 *Q_y_* absorption above 900 nm to 915 nm and significantly enhance its thermal stability. In the mesophilic purple bacterium *Thiorhodovibro frisius*, its LH1−RC complex [7] also forms a closed 16-subunit LH1 containing 16 Ca^2+^. However, in this case, Ca^2+^ binding-induced hydrogen bonding networks induce an extremely large redshift, making the *Trv. frisius* LH1 is the most redshifted of all known BChl *a*-containing LH1 complexes. Similar to *Tch. tepidum* and *Trv. frisius*, the LH1 complex from the mildly thermophilic purple sulfur bacterium *Allochromatium* (*Alc.*) *tepidum* [15] also contains Ca^2+^, and this metal has been shown to affect both its *Q_y_* transition and thermal stability [16,17]. A cryo-EM structure of the *Alc*. *tepidum* LH1−RC complex [14] revealed that the LH1 component contains three forms of α-polypeptides and two forms of β-polypeptides encoded by three pairs of *pufBA* genes (*pufB*_1_*A*_1_, *pufB*_1_*A*_2_, and *pufB*_3_*A*_3_); this is also true of its closely related mesophilic counterpart *Alc. Vinosum* [18]. The ratio of α1:α2:α3 per complex is 6:9:1, and of β1:β3 it is 10:6, respectively. Among these αβ-heterodimers, only those formed by α1 with β1 or β3 can bind calcium ions due to the calcium-ion binding WxxDxI motif existing in α1 but not in α2 or α3; therefore, only six calcium ions are present in the LH1 complex of *Alc*. *tepidum* and *Alc. vinosum*. These partially Ca^2+^-bound complexes likely control the unusual thermostability and spectral properties of these two species. Their characteristic arrangement of multiple αβ-polypeptides has also hinted that a molecular mechanism must exist for recognizing, expressing, and assembling the LH1 complex, a mechanism that could be regulated through interactions between LH1 and RC subunits [14,18].

Motivated by these observations, here we have investigated the assembly of αβ-polypeptides and the influence of Ca^2+^ on modified forms of the *Alc. tepidum* LH1 complex. A previously developed *Rhodospirillum* (*Rsp.*) *rubrum* mutant strain [19,20] was employed to construct and express Ca^2+^-bound and Ca^2+^-free *Alc. tepidum* LH1-only complexes composed of different α- and β-polypeptides either containing or lacking the Ca^2+^-binding motif. Key biochemical properties of these heterologously expressed LH1-only photocomplexes were then characterized, and their structures were determined. Unlike native LH1 complexes, each of the LH1-only complexes contained a homogeneous α/β-apoprotein composition and, as such, facilitated experiments to reveal how specific α/β combinations affect spectral tuning and thermal stability.

## 2. Materials and Methods

### 2.1. Strains and Growth Conditions

The strains used in this study are those described elsewhere with minor modifications [14]. *Rsp*. *rubrum* strain H2, which naturally lacks LH2, was employed as the host cell, and this photosynthetically incompetent mutant strain was derived from wild-type *Rsp*. *rubrum* by deleting the *puhA* and *pufBALM* genes (collectively encoding its LH1–RC) [19]. The cells were grown at 30 °C in a modified Tryptic Soy Broth (TSB) medium supplemented with rifampicin. When the cell cultures reached an OD_680nm_ of 1.2–1.5, aliquots were transferred to Erlenmeyer flasks filled to 80% capacity and shaken at 200 rpm for 24 h in darkness; under these conditions, heterologous membrane protein production was induced. *E*. *coli* strain WM3064 was grown in an LB medium supplemented with DL-2,6-diaminopimelic acid.

### 2.2. Construction of Rsp. rubrum Mutants That Biosynthesized Alc. tepidum LH1-Only Complexes

The genes *pufB1A1* (0.415 kb), *pufB1A2* (0.355 kb), and *pufB3A1* (0.419 kb) of *Alc. tepidum* was synthesized artificially (Tianyihuiyuan Bioscience and Technology Co., Ltd., Beijing, China). These genes were then combined with the purified 1.2 kb upstream fragment of the *pufBA* gene and the 1.3 kb downstream fragment of the *pufLM* gene and inserted into the suicide vector pJQ200SK using the In-Fusion HD Cloning Kit (Mei5 Biotechnology Co., Ltd., Beijing, China). The constructs were subsequently cloned into *E. coli* WM3064 and transferred into *Rsp*. *rubrum* H2 via conjugation as described elsewhere [21]. Transformants were screened for the desired constructs using 50 μg/mL each of gentamicin and rifampicin, followed by selection for sucrose resistance. Positive strains were confirmed by PCR and DNA sequencing. A list of the PCR primers used in this study is present in Figure S1B.

### 2.3. Purification of Alc. tepidum LH1-Only Complexes

Cell culture and protein purification of the *Alc. tepidum* LH1-only complex was performed following previous protocols with slight modifications [20]. Cells were grown in darkness up to the mid-exponential phase. The *Rsp. rubrum* mutant cells expressing the *Alc. tepidum* LH1-only complexes were harvested and suspended in 20 mM Tris-HCl buffer (pH 7.5). The cells were disrupted by sonication (Q700 Sonicator, Qsonica, Newtown, PA, USA) in an ice-water bath with an output power of 50 W, applied in cycles of 3 s on and 5 s off. The total sonication time was 20 min, ensuring the cell suspension remained thoroughly cooled throughout the process. Chromatophores were isolated by ultracentrifugation at 200,000× *g* at 4 °C for 60 min. For Ca^2+^-bound LH1-α1β1 and LH1-α1β3 complexes, a two-step solubilization method was performed. The chromatophores were first solubilized with 1.0% *n*-octyl-β-D-glucopyranoside (β-OG) for 60 min at room temperature followed by ultracentrifugation at 200,000× *g* at 4 °C for 60 min; the resulting membranes were then solubilized with 1.0% *n*-dodecyl-β-D-maltopyranoside (β-DDM) for 60 min at room temperature followed by ultracentrifugation at 200,000× *g* at 4 °C for 60 min. For the Ca^2+^-free LH1-α2β1 complex, treatment with 1.0% β-DDM for 60 min at room temperature was followed by ultracentrifugation at 200,000× *g* at 4 °C for 60 min, solubilizing most of the LH1-α2β1 complexes. In all cases, the LH1-only containing supernatants were then loaded onto a DEAE-650S column equilibrated with 20 mM Tris-HCl (containing 0.05% β-DDM, pH 7.5) at room temperature. Fractions were eluted with linear gradients of NaCl (0–50 mM), and the peak fractions with ratios of *A*_894_/*A*_280_ ≥ 2.7 for Ca^2+^-bound complexes and *A*_888_/*A*_280_ ≥ 2.7 for Ca^2+^-free, respectively, were collected for biochemical characterization and structural analyses. Purity and homogeneity of the reconstituted *Alc. tepidum* LH1-only complexes were assessed by SDS-PAGE and negative stain electron microscopy (Figure S2).

### 2.4. Spectroscopy and Thermal Stability

Absorption spectra were recorded on a SHIMADZU UV-1900i (SHIMADZU, kyoto, Japan) spectrophotometer using quartz semi-micro cuvettes with a wavelength range of 250–1000 nm. The concentrations of three LH1-only complexes were adjusted to a *Q_y_* OD of about 1.5, and EDTA titration experiments were conducted by the addition of EDTA to purified samples to a final concentration of 50 mM followed by the addition of CaCl_2_ to a final concentration of 175 mM. An interval of five min between additions of EDTA and CaCl_2_ was taken to allow a more complete recovery of the *Q_y_* band.

To assess thermal stability, LH1 preparations were adjusted to 1 mg/mL in buffer containing 20 mM Tris-HCl (pH 7.5) and 0.03% (*w*/*v*) β-DDM. Thermal stability of the samples was recorded as *Q_y_* band intensities after incubation for 5 min at 65–75 °C and at a given temperature for 0–96 min. DSC measurements were recorded on a nanoDSC II calorimeter (TA, Newtown, PA, USA). Thermal degradation was monitored at a range of 30–100 °C at a heating rate of 1 °C/min.

### 2.5. Cryo-EM Data Collection

Proteins for cryo-EM analysis were concentrated to ~5 mg/mL. Three microliters of the protein solutions were applied on a glow-discharged holey Nitinol grid (Amorphous alloy film R1.2/1.3, 300 mesh, Nanodim) that had been treated with Ar and O_2_ mixtures in a Solarus plasma cleaner (Gatan, Pleasanton, CA, USA) for 100 s and then blotted and plunged into liquid ethane at –182 °C using an Vitrobot Mk4 plunger (Leica, Microsystems, Vienna, Austria). The applied parameters were a blotting time of 4 s at 100% humidity and 4 °C. Data were collected on a Titan Krios (Thermo Fisher Scientific, Hillsboro, OR, USA) electron microscope at 300 kV equipped with a K3 camera (Thermo Fisher Scientific). Movies were recorded using EPU software 2.12.0.2771 (Thermo Fisher Scientific) at a nominal magnification of 81 K in counting mode and a pixel size of 1.04 Å with a CDS mode corresponding to 1.58 e^−^ per Å^2^ per second at the specimen level. Each movie included 40 fractioned frames, resulting in an accumulated dose of 60.0 e^−^ per Å^2^ (Table 1).

### 2.6. Image Processing of LH1-α1β1 and LH1-α2β1 Complex

All stacked frames were subjected to patch motion correction with cryoSPARC [22]. Defocus was estimated by patch CTF estimation. Of total particles, 1,882,697 and 863,338 were auto-picked by crYOLO [23] for LH1-α1β1 and LH1-α2β1, respectively, using a pre-trained model and further selected by 2D classification. After two rounds of 2D classification, 298,981 and 322,558 good particles for LH1-α1β1 and LH1-α2β1, respectively were sorted out for 3D reconstruction (ab-initio). All particles were subjected to a lowpass filter of 3 Å before reconstruction. Four initial 3D models were generated by ab-initio reconstruction followed by heterogeneous refinement (Figures S3A and S4A). For LH1-a1b1, the best model corresponding to 234,287 particles was selected for 3D refinement, while for LH1-α2β1, the best model corresponding to 239,622 particles was selected for further classification. Further heterogeneous refinement removed 32,846 poor particles to produce the final dataset containing 206,784 particles for LH1-α2β1. Preliminary NU-refinement [24] was performed without any symmetry imposed, and the low-pass filtered particles resulted in maps of 2.54 Å resolution for LH1-α1β1 and 3.28 Å resolution for LH1-α2β1. The raw particles were then re-extracted for NU-refinements with CTF refine and C14 symmetry applied, resulting in the final maps with resolutions of 2.45 Å for LH1-α1β1 and 2.78 Å for LH1-α2β1 according to the gold-standard Fourier shell correlation (FSC) using a criterion of 0.143 (Figure 3, Figures S3A and S4A). Local resolution maps were calculated by the cryoSPARC’s built-in local resolution estimation tool.

### 2.7. Model Building and Refinement of the LH1-Only Complex

The atomic model of the LH1 complex from *Alc. tepidum* (PDB: 7VRJ) was selected and altered to that only containing α1β1 and α2β1 using SWISS-MODEL [25] and then fitted to the cryo-EM map obtained for the *Alc. tepidum* LH1-only using Chimera [26]. Further manual adjustment and real space refinement for the polypeptides and cofactors were performed using COOT [27]. The manually modified model was refined in real space on PHENIX [28], and the COOT/PHENIX refinement was iterated until the refinements converged. Finally, the statistics calculated using MolProbity were checked. Figures were drawn using the Pymol Molecular Graphic System [29], UCSF Chimera, and ChimeraX.

## 3. Results

### 3.1. Construction, Purification, and Characterization of Heterologously Expressed Alc. tepidum LH1-Only Complexes

Mutant strain H2 of the LH2-lacking species *Rsp. rubrum*, which in addition lacks the LH1-RC complex was used to construct a genetic system. The *Alc. tepidum pufBA* (*pufB*_1_*A*_1_, *pufB*_1_*A*_2_, *pufB*_3_*A*_1_) genes were integrated into the genome of *Rsp. rubrum* H2 for synthesizing the LH1 α/β-polypeptides (Figure S1), and the corresponding photocomplexes were named LH1-α1β1, LH1-α2β1 and LH1-α1β3, respectively. Heterologously expressed LH1 polypeptides were then successfully assembled in strain H2 into membrane-associated pigment–protein complexes containing BChl *a* and carotenoids as confirmed by absorption spectra of intracytoplasmic membranes (ICM) (Figure S1) in which the characteristic absorption bands of BChl *a* and carotenoids are similar to that of native *Alc. tepidum* LH1−RC complex.

**Figure 1:**
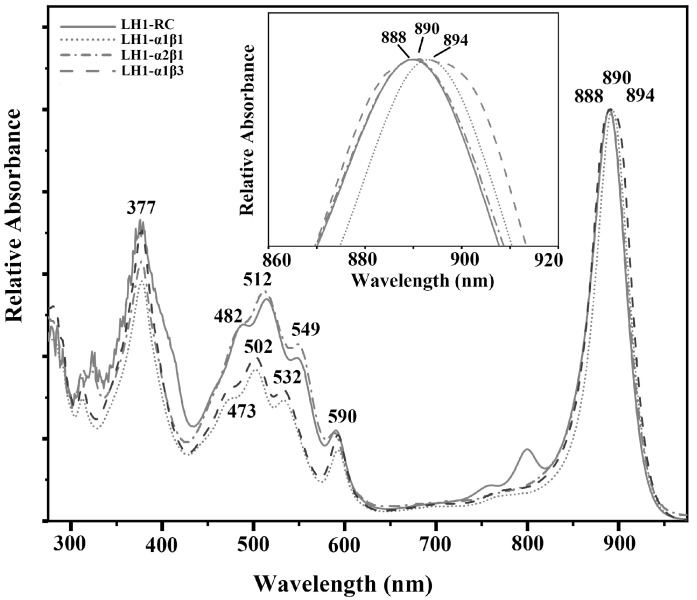
Comparison of absorption spectra of the purified heterologously expressed *Alc. tepidum* LH1-only complexes with native LH1−RC complex. Inset, the expanded view of the LH1 *Q_y_* region. Curve pattern: LH1-α1β1 (dotted line), LH1-α2β1 (dash-dotted line), LH1-α1β3 (dashed line), and native LH1−RC complex (solid line).

Figure 1 shows the absorption spectrum of purified *Alc. tepidum* LH1-only complexes produced in *Rsp. rubrum* H2. It is clear that the 800 and 761 nm bands due to accessory BChl *a* and bacteriopheophytin *a* (BPhe) in the RC are missing, thus confirming the absence of the RC in the purified LH1 complexes. The *Q_y_* transition of the LH1-α1β1 and LH1-α1β3 complex (both containing the Ca^2+^-binding motif) occurred at 894 nm, whereas the *Q_y_* of the LH1-α2β1 complex lacking a Ca^2+^-binding motif was at 888 nm. Compared with the native *Alc. tepidum* LH1−RC, whose *Q_y_* transition lies at 890 nm, these complexes showed a slight redshift and blueshift, respectively. Besides these shifts in the LH1-*Q_y_* transition, another change was observed in the carotenoid absorption. The spectrum of the Ca^2+^-free LH1-α2β1 complex was similar to that of the native LH1−RC, while both the intensity and peaks of carotenoid absorption in Ca^2+^-bound LH1-only complexes differed. In the latter, peaks at 473, 502 and 532 nm showed respective blue shifts of 9, 10 and 17 nm as compared with that of the native LH1−RC, signaling that the carotenoid composition changed during biosynthesis and assembly of these complexes. These results were confirmed by pigment analysis that indicated the main carotenoids were spirilloxanthin and anhydrorhodovibrin in Ca^2+^-free and Ca^2+^-bound LH1-only complexes, respectively (Table 2).

Carotenoids are listed in the order in which they are produced biosynthetically, starting from lycopene [30].

**Figure 2:**
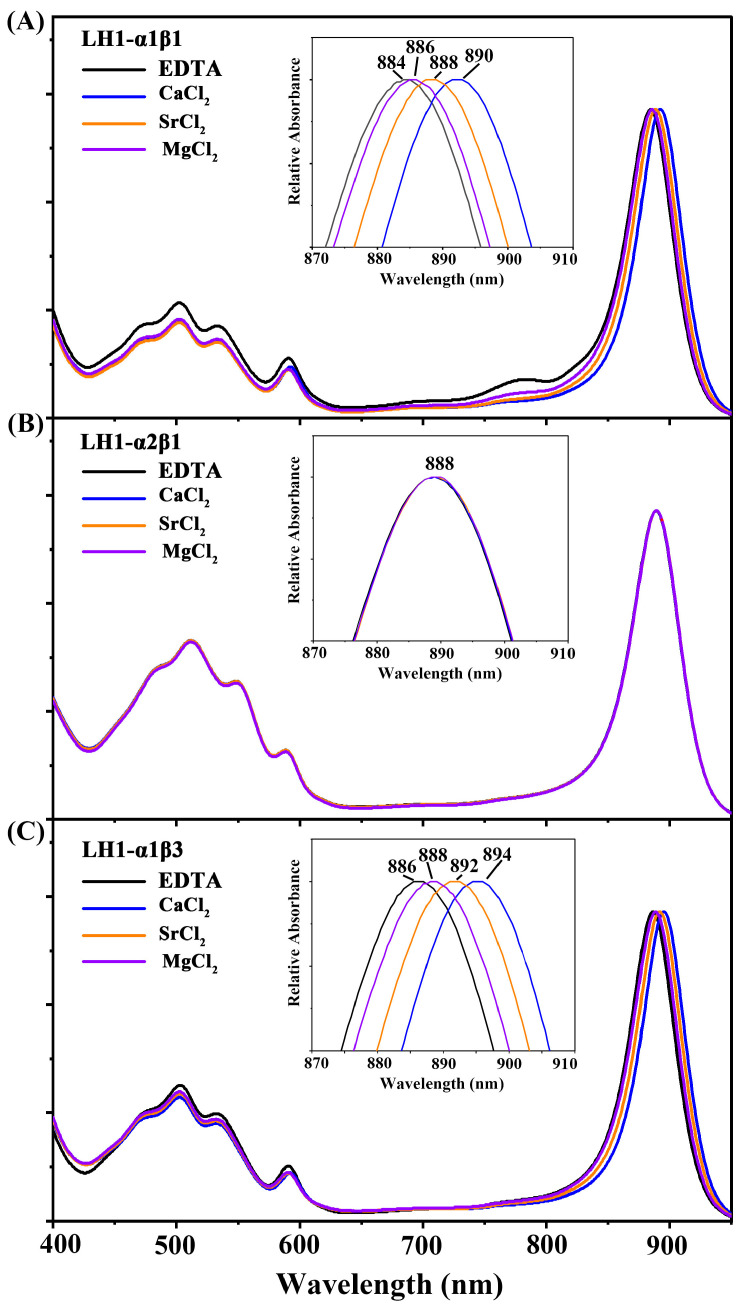
Spectral changes of the *Alc. tepidum* LH1-only complexes induced by the addition of EDTA and various cations (100 mM). From top to bottom, LH1-α1β1 (**A**), LH1-α2β1 (**B**) and LH1-α1β3 (**C**). Inset, the expanded view of the LH1 *Qy* region. Curve color: EDTA (black), CaCl_2_ (blue), SrCl_2_ (orange), MgCl_2_ (purple).

Figure 2 illustrates the effects of EDTA on LH1-*Q_y_* transitions of the different LH1-only complexes and their spectral changes under different conditions. In the presence of EDTA, the *Q_y_* band of Ca^2+^-bound LH1-α1β1 and LH1-α1β3 complex blueshifted by ~10 nm from 894 nm to 884–886 nm, and the addition of Ca^2+^ recovered the *Q_y_* to 894 nm, similar to results with native *Alc. tepidum* LH1−RC (reversible shifting from 890 to 882 nm) [16]. Interestingly, the addition of Sr^2+^ or Mg^2+^ partially restored the *Q_y_* redshift (Figure 2A,C), similar to results obtained with these alternatives to Ca^2+^ in *Tch. tepidum* [31]. Collectively, these data indicate that Ca^2+^-containing *Alc. tepidum* LH1-only complexes exhibit nearly identical spectral characteristics to the native LH1 complex, and the Ca^2+^-dependent redshift of the LH1-*Q_y_* transition is independent of (or unaffected by) the RC complex. In contrast to the Ca^2+^-bound complexes, and as expected, *Q_y_* absorption of Ca^2+^-free complexes was entirely impervious to the influence of calcium ions, EDTA, or alternative metal ions (Figure 2B).

### 3.2. Structures of Chimeric Alc. tepidum Ca^2+^-Bound and Ca^2+^-Free LH1-Only Complexes

**Figure 3:**
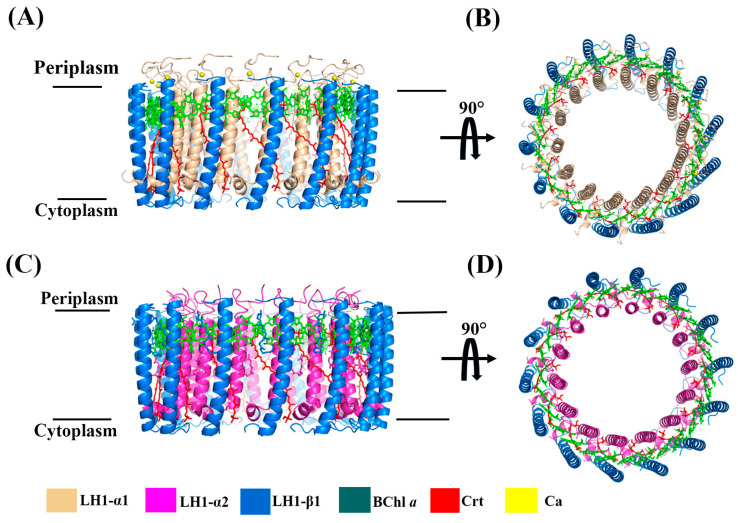
Overall structure and cofactor arrangement of the *Alc. tepidum* LH1-only complexes. (**A**, **C**) Side view of the LH1-α1β1 and LH1-α2β1 complex parallel to the membrane plane. (**B**, **D**) Top view of the LH1-α1β1 and LH1-α2β1 complex from the periplasmic side of the membrane. The phytol tails were omitted for clarity. Color scheme: LH1-α1, wheat; LH1-α2, magenta; LH1-β1, blue; BChl *a*, green; Crt, red; Ca^2+^, yellow.

**Figure 4:**
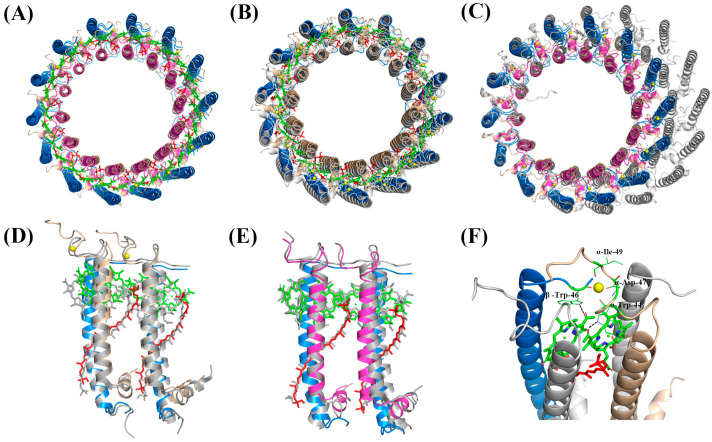
Structural details of the *Alc. tepidum* LH1-only complexes. (**A**) Comparison of the overall structure of LH1-α1β1 and LH1-α2β1 complex. (**B**) Comparison of the overall structure of LH1-α1β1 complex and the *Tch*. *tepidum* LH1-only complex (PDB: 8JC9). (**C**) Comparison of the LH1-only complex with the native LH1–RC complex (RC and cofactors are omitted for clarity) colored in gray from *Alc*. *tepidum* (PDB: 7VRJ). (**D**, **E**) Comparison of the LH1 subunit (α1β1 and α2β1) between the LH1-only complex and those in the native LH1–RC complex (colored in gray). (**F**) Ca^2+^-binding site in the LH1-α1β1 complex. The Ca^2+^ coordinating residues are labeled, and the hydrogen bonds between BChl *a* and tryptophan are shown as dashed lines. Color scheme as in Figure 3.

Due to the high sequence similarity between *Alc. tepidum* LH1 β3 and β1 [14] and the similar absorption spectra observed for purified LH1-α1β1 and LH1-α1β3 complexes (which are markedly different from that of LH1-α2β1), it is inferred that LH1-α1β1 and LH1-α1β3 should also have similar structures and pigment contents. Thus, LH1-α1β1 and LH1-α2β1 were used as models of the Ca^2+^-bound and Ca^2+^-free complexes, respectively, and their cryo-EM structures were determined at resolutions of 2.45 and 2.78 Å, respectively (Figure 3 and Figures S3–S5). The overall structures of these two complexes were similar—both were circular assemblies of 14 αβ-heterodimer subunits, two pairs fewer than the more elliptical-shaped native *Alc. tepidum* LH1−RC complex [14]; as such, the circular topology of these LH1-only complexes would not be able to accommodate the RC (Figure 4C). In both complexes, each αβ-heterodimer subunit bound two molecules of BChl *a* and one carotenoid. In the Ca^2+^-containing LH1 (α1β1) complex, 14 Ca^2+^ were present on the periplasmic side of the membrane, and the details of calcium ion coordination are shown in Figure 4F. Ca^2+^ is coordinated by Trp44, Asp47, and Ile49 of α1 and Trp47 of the β-polypeptide. By contrast, in the Ca^2+^-free LH1-α2β1 complex, the regions corresponding to Ca^2+^-binding sites remained empty, consistent with the Ca^2+^ binding pattern in the native *Alc. tepidum* LH1−RC, further validating the authenticity of the Ca^2+^-binding motif WxxDxI (Figure 4F).

Apart from the presence or absence of Ca^2+^ binding sites, the sequence similarity between α2 and α1 is only 57%, significantly lower than the high similarity between β3 and β1 (80% homology). However, the overall structures of both LH1 (α1β1) and LH1 (α2β1) complexes are still quite similar, with the main difference residing at the C-terminal region due to the longer sequence of α1 compared with α2 (Figure 4A). Comparing reconstructed LH1 complexes composed of α1β1 or α2β1 heterodimers with the native LH1−RC complex showed the arrangement of pigments to be virtually the same and that the αβ heterodimers deviated only slightly, indicating that the *Q_y_* absorption was not affected by the deviation of these polypeptides.

### 3.3. Effects of Ca^2+^ on Thermostability of Chimeric Alc. tepidum LH1-Only Complexes

**Figure 5:**
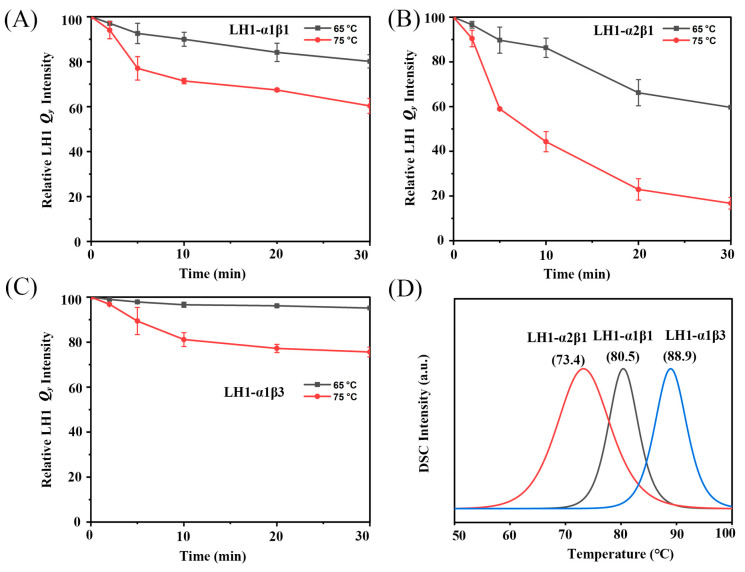
Changes in the relative LH1 *Q_y_* intensities of LH1-only complex at different temperatures and DSC scans. Changes in the relative LH1 *Q_y_* absorption intensities of *Alc. tepidum* LH1-α1β1 (**A**), LH1-α2β1 (**B**), and LH1-α1β3 (**C**) at different temperatures as functions of time. (**D**) DSC scans of three kinds of LH1-only complexes. The concentration was 1 mg/mL, and the buffer contained 20 mM Tris-HCl (pH 7.5) and 0.03% β-DDM. Scan rates were 1 °C per min.

Given the moderate thermophilicity of *Alc*. *tepidum* [15] and the thermal stability observed in our previous work with the *Tch*. *tepidum* LH1-only complex [20], we further investigated the thermal stability of Ca^2+^-bound and Ca^2+^-free LH1-only complexes at temperatures of 65 °C and 75 °C. Figure 5A−C shows absorption changes of the two Ca^2+^-bound *Alc*. *tepidum* LH1 complexes and a Ca^2+^-free LH1-only complex over time at the two temperatures. The Ca^2+^-bound LH1-only complexes exhibited significantly higher thermal stability, especially for LH1-α1β3, whose *Q_y_* band intensity was essentially unchanged for up to 30 min at 65 °C. In contrast, the *Q_y_* band of the Ca^2+^-free LH1-α2β1 complex showed a marked decrease, retaining only about 60% of its LH1 *Q_y_* intensity at 65 °C. At 75 °C, the trend was even more pronounced, with the relative intensity of the Ca^2+^-free LH1 *Q_y_* band decreasing by 40 and 80 % at the two temperatures, respectively (Figure 5B). Among the three complexes, the Ca^2+^-bound LH1-only complexes demonstrated markedly higher thermal stability than the Ca^2+^-free counterpart, and within the Ca^2+^-bound LH1-only complexes, the LH1-α1β3 complex exhibited greater thermal stability than did the LH1-α1β1 complex (Figure 5A,C). These data clearly indicate that Ca^2+^ is essential for the thermal stability of the *Alc*. *tepidum* LH1–RC complex, but also that different α–β combinations confer different degrees of heat stability.

The thermal stability of LH1-only complexes was further quantified by differential scanning calorimetry (DSC). Figure 5D displays the endothermic profiles of Ca^2+^-bound and Ca^2+^-free LH1-only complexes. For the Ca^2+^-bound LH1-α1β1 and LH1-α1β3 complexes, a major peak was observed at 80.5 °C and 88.9 °C, respectively. Interestingly, their denaturing temperatures were markedly higher than that of the native *Alc. tepidum* LH1 bound to the RC in the intact LH1−RC complex (the latter denatures at 75.7 °C) [16]. For the Ca^2+^-free LH1-α2β1 complex, the denaturing temperature was markedly decreased to 73.4 °C, slightly lower than the native LH1−RC complex. In summary, these results suggest that a heat stability hierarchy exists: LH1-α3β1 > LH1-α1β1 > LH1-α2β1 and underscore the importance of Ca^2+^ for the thermostability and structural integrity of the *Alc*. *tepidum* core photocomplex.

## 4. Discussion

It is not uncommon to see multiple antenna genes encoding the LHC complexes of photosystem I and photosystem II of plants and algae or the LH2 and LH1 complexes of purple bacteria. For example, in the latter, multiple genes encoding LH1 and LH2 complexes allow the organism to adapt to changing light conditions. Similarly, in plants, LHC gene families such as *lhcb* and *lhca* encode multiple forms of LHC proteins specialized for different light-harvesting roles in photosystem II and photosystem I, respectively. This specialization enables fine-tuning of light absorption and energy transfer efficiencies under varying environmental conditions. However, the reason why these organisms contain redundant genes is sometimes unclear. For bacterial LH complexes, it is possible that redundant genes may be necessary for environmental adaptation, utilizing minor sequence changes in LH polypeptides to bind different pigment molecules and/or cofactors in response to changing environmental conditions in light quality/intensity or temperature. Due to the simplicity of their photosynthetic molecular apparatus compared with that of oxygenic phototrophs, coupled with their facile molecular genetics, phototrophic purple bacteria have long been ideal models for studying these important problems. Recently, redundant *pucBA* genes, which encode LH2 in the purple nonsulfur bacteria *Rhodopseudomonas palustris* and *Rhodobacter sphaeroides*, were knocked out to obtain an LH2 complex encoded by a single *pucBA* [5,32]. The latter was then used for structural analyses, providing a more focused model for investigating the relationship between structure and function in the peripheral antenna system [5]. However, similar studies on LH1−RC complexes have not yet been reported.

The purple phototrophic bacterium *Alc*. *tepidum* has been considered an “intermediate” species between mesophilic and thermophilic purple sulfur bacteria in the sense that its LH1−RC complex is moderately thermal stable and displays only moderate redshift (compared with that of *Tch. tepidum*) in *Q_y_* absorption from the binding of six Ca^2+^ within the complex [14]. However, because molecular genetics and expression systems in both *Alc. tepidum* and its more thermophilic relative *Tch. tepidum* have not been developed, genetic approaches thus far have relied on surrogate hosts. In this connection, *Rsp. rubrum* strain H2, which naturally lacks LH2, has been quite useful for probing the intrinsic properties of LH1 complexes containing multiple αβ polypeptides and the roles of cofactors such as Ca^2+^. Based on the previously reported cryo-EM structure of *Alc. tepidum* [14], we were able to construct three hybrid LH1 complexes here—LH1-α1β1, LH1-α2β1, and LH1-α1β3—in strain H2 and express and purify them for biochemical and structural studies.

Characteristic *Q_y_* absorption peaks of BChl *a* in the different complexes were centered around 890 nm, confirming the expression and assembly of these polypeptides in host cells. Among them, the *Q_y_* absorption peaks of Ca^2+^-bound LH1-α1β1 and LH1-α1β3 occurred at 894 nm, while the Ca^2+^-free LH1-α2β1 complex exhibited a *Q_y_* peak at 888 nm (the native LH1 *Q_y_* absorption peak in the *Alc. tepidum* LH1–RC complex is at 890 nm [16], positioned between the bands of the Ca^2+^-containing and Ca^2+^-free LH1 complexes). The spectral properties of light-harvesting complexes are influenced by two structural factors. First, the distances and orientations between pigments dictate the extent of exciton coupling, facilitating excited-state delocalization, band splitting, and redistribution of dipole strengths across various states. And second, the composition and arrangement of the surrounding environment alter the site energy of pigments through solvatochromic effects, ultimately causing shifts in absorption bands [33]. In the LH1 complexes studied here, the protein environment surrounding BChl is highly similar, and the comparison between complexes with or without Ca^2+^ implies a direct ion effect on the *Q_y_* transition of 6 nm. The broader absorption band observed in the LH1-α1β3 complex (compared to the more defined peak in the LH1-α1β1 complex) could be due to differences in the pigment environments within the complexes. Specifically, the LH1-α1β3 complex may have a more dynamic or less rigid pigment environment, which could lead to a broader absorption feature.

An apparent difference in carotenoid composition, indicating the impact of carotenoid biosynthesis enzymes on the heterologous expression process, was also observed in the absorption spectra. Pigment analysis revealed that the complexes contained different complements of carotenoids. In the Ca^2+^-free LH1-α2β1, the major carotenoid was spirilloxanthin (C_42_), as is true of the native *Alc. tepidum* LH1-RC complex. By contrast, the Ca^2+^-bound LH1-α1β1 and LH1-α1β3 complexes contained mainly anhydrorhodovibrin (C_41_), a precursor of spirilloxanthin. It has been reported that carotenoids play a significant role in stabilizing photosynthetic complexes and, in some species, may confer thermostability on photocomplexes [34]. Therefore, the differences between LH1-α1β1 and LH1-α2β1 complex in carotenoid components may affect the thermal stability of their proteins. Alternatively, considering the protein sequence differences between LH1-α1β1 and LH1-α1β3, other interpretations are possible; for example, that the observed differences in thermal stability are simply the result of sequence-based structural variations.

Cryo-EM analysis revealed that the overall structures of both Ca^2+^-bound and Ca^2+^-free *Alc. tepidum* complexes were quite similar, and although the types of bound carotenoids differed slightly, their spatial positions were essentially the same. Comparison of LH1-α1β1 and LH1-α2β1 with the α1β1 and α2β1 heterodimer present in native *Alc. tepidum* LH1–RC complexes showed that these structures are virtually the same, implying that it should be feasible to assemble a native core complex with a single form of αβ heterodimer in the presence of the RC, both for the Ca^2+^-bound and Ca^2+^-free forms. However, the formation in both of smaller 14-subunit ring structures indicates a more stable state than native 16-subunit structures in the absence of RC. Assuming this is true raises the intriguing question of why the native *Alc. tepidum* LH1–RC complex contains multiple forms of αβ. In the native LH1–RC, there are three forms of α (α1, α2, and α3) and two forms of β (β1 and β3) of varying stoichiometries, and among them, β-polypeptides show a higher similarity to each other than α-polypeptides do to each other [14]. This may be related to the reduced function of β-polypeptides, which only participate in binding sites for BChl and Ca^2+^ in contrast to the dual role of α-polypeptides in both binding BChl and interacting selectively with surrounding RC subunits.

Similar to previous results with LH1-only complexes from *Tch*. *tepidum* [20], all three *Alc*. *tepidum* LH1-only complexes exhibited enhanced thermal stability. However, in contrast to what was found in *Tch. Tepidum*, where a mixture of 14- and 15-subunit LH1-only complexes were detected, all *Alc*. *tepidum* LH1-only complexes contained 14 subunits. It is noteworthy that among the three *Alc*. *tepidum* complexes constructed, the thermal stability of both Ca^2+^-bound complexes was comparable and distinctly better than that of the Ca^2+^-free complex, further confirming that enhanced thermal stability of the complex arises primarily from bound Ca^2+^. The organization of αβ polypeptides within the LH1 ring points to a highly regulated mechanism governing the expression and assembly of the *Alc*. *tepidum* LH1 complex, particularly as regards the location of specific forms of αβ polypeptides. Moreover, although structural analyses of LH1–RC complexes from different purple bacteria have revealed that LH1s can be composed of as few as 10 or as many as 24 subunits, the rationale for this major difference also remains unclear. Although LH1 protein sequences and structures are relatively highly conserved across different purple bacteria, the factors driving the synthesis of different forms of αβ polypeptides and the mechanisms that regulate their assembly remain unknown. The *Rsp. rubrum* cloning and expression system employed herein provides a powerful tool for exploring these important questions, such as the detailed molecular mechanisms underlying the assembly and function of a diverse LH1–RC complex, with a particular focus on the role of other potential regulatory factors in addition to Ca^2+^. This could involve mutagenesis studies and functional assays to identify key residues and interactions involved in complex formation and stability. Coupled with the structural insight available from cryo-EM, the combination of molecular genetics and structural biology should provide a clearer picture of mechanisms that govern the assembly of photocomplexes and may uncover specific modifications that support the ecological success of specific organisms.

## 5. Conclusions

This research demonstrates that the presence of Ca^2+^ plays a pivotal role in modulating the structure, stability, and spectral properties of the light-harvesting 1 (LH1) complex in *Alc. tepidum*, a mildly thermophilic purple sulfur bacterium. Through the comparison of Ca^2+^-bound and Ca^2+^-free LH1 complexes expressed in *Rsp. rubrum*, distinct differences in their *Q_y_* absorption maxima were observed, with a 6 nm red shift induced by Ca^2+^ binding. Furthermore, Ca^2+^-bound LH1 complexes exhibited enhanced thermostability, highlighting the crucial function of Ca^2+^ in stabilizing the photocomplex and modulating its spectral characteristics. These findings provide new insights into the functional adaptability of LH1 complexes in thermophilic organisms and underscore the importance of Ca^2+^ in the ecological success of *Alc. tepidum*.

## Figures and Tables

**Table 1:** Cryo-EM data collection, refinement, and validation statistics.

	LH1-α1β1 Complex	LH1-α2β1 Complex
	(EMD-39475)	(EMD-39477)
	(PDB ID 8YPB)	(PDB ID 8YPD)
Data collection and processing		
Magnification	81,000	81,000
Voltage(kV)	300	300
Electron exposure(e-/Å^2^)	60	60
Defocus range(μm)	−0.8~−2.5	−0.8~−2.5
pixel size(Å)	1.04	1.04
Symmetry imposed	C14	C14
Initial particle images (no.)	1,882,697	863,338
Final particle images (no.)	234,287	206,784
Map resolution (Å)	2.45	2.78
FSC threshold	0.143	0.143
Refinement		
Initial model used (PDB code)	5Y5S	5Y5S
Model resolution (Å)	2.5	2.8
FSC threshold	0.5	0.5
Map sharpening B factor (Å^2^)	90.7	108.2
Model composition		
Non-hydrogen atoms	13,286	12,292
Protein residues	1330	1190
Ligands	56	42
B factors (Å^2^)		
Protein	11.18	15.41
Ligand	7.54	7.65
R.m.s.deviations		
Bond lengths (A)	0.008	0.008
Bond angles (°)	1.236	1.208
Validation		
MolProbity score	1.89	1.59
Clashscore	14.01	12.06
Poor rotamers (%)	2.03	0.49
Ramachandran plot		
Favored (%)	99.37	100.00
Allowed (%)	0.63	0.00
Disallowed (%)	0.00	0.00

**Table 2:** Carotenoid composition (mol % of total carotenoids) in LH1-α1β1, LH1-α2β1, LH1−RC, and whole cell of *Alc. tepidum*.

Carotenoid	LH1-α1β1	LH1-α2β1	Cell	LH1−RC
Lycopene	nd	nd	4.3	0.4
Rhodopin	14.63	nd	57.4	7.4
3,4,3′,4′-Tetrahydro spirilloxanthin	nd	21.65	nd	nd
Anhydrorhodovibrin	58.46	11.12	13.7	6.1
Rhodovibrin	14.64	11.53	2.2	4.7
OH-spirilloxanthin	nd	nd	nd	nd
Spirilloxanthin	12.27	55.70	16.5	73.2

Values in each column are the percent of total carotenoids in the listed structure; nd, not detected. Data for *Alc. tepidum* NZ cell and LH1−RC complex obtained from Kimura [15,16].

## Data Availability

Cryo-EM density maps were deposited in the Electron Microscopy Data Bank (EMDB, www.ebi.ac.uk/pdbe/emdb/) under the following accession codes: EMD-39475 for Ca^2+^-bound LH1-only complex and EMD-39477 for Ca^2+^-free LH1-only complex. The atomic coordinates have been deposited in the Protein Data Bank (PDB, www.rcsb.org) under the following accession codes: 8YPB and 8YPD. All other data are available from the corresponding authors upon reasonable request.

## Supplementary Materials

The following supporting information can be downloaded at https://www.mdpi.com/article/10.3390/biom15010124/s1, Figure S1: Heterologous expression of the *Alc. tepidum* LH1-only complex; Figure S2: Characterization of the *Alc. tepidum* LH1-only complexes obtained in this study; Figure S3: Cryo-EM data process of the *Alc. tepidum* LH1-α1β1 complex; Figure S4: Cryo-EM data process of the *Alc. tepidum* LH1-α2β1 complex; Figure S5: Cryo-EM densities and structural models of the polypeptides in the *Alc. tepidum* LH1-only complexes; Figure S6: Changes in the absorption spectra on thermal degradation at 65 °C (A) and 75 °C (B) for the *Alc. tepidum* LH1-only complexes.
