# Supplementary material for: Probing the Dual Role of Ca2+ in the Allochromatium tepidum LH1–RC Complex by Constructing and Analyzing Ca2+-Bound and Ca2+-Free LH1 Complexes
Source: Biomolecules. 2025 Jan 14;15(1):124. doi: 10.3390/biom15010124 (PMC11764377; doi:10.3390/biom15010124)
Supplement: Supplementary file 1 [file biomolecules-15-00124-s001.zip › biomolecules-3248095-supplementary.pdf]

(A)

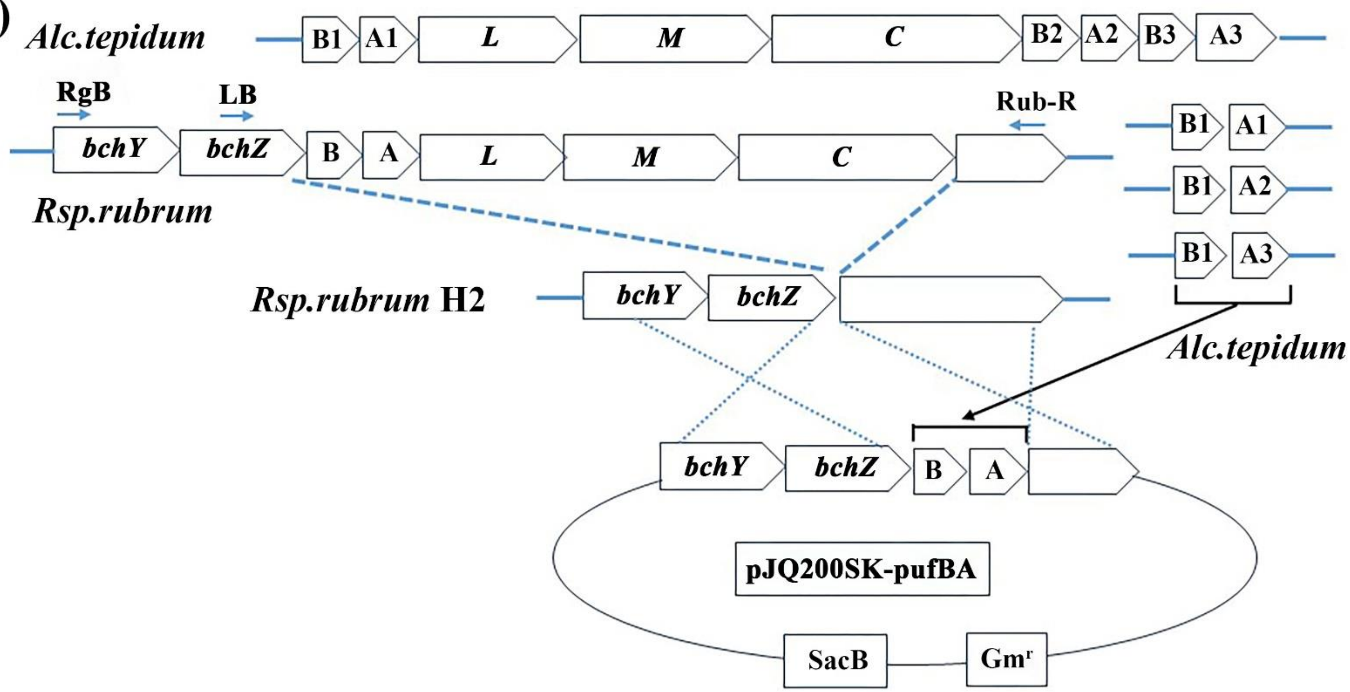

(B)

Primer name	Sequence (5' - 3')
RgB	CCATCGACGAGGACCAGTGGCAGAG
LB	GTTATCCGCCTGCTCGATCTTC
Rub-R	AGGCTATGCGATCAAGCTCAGGC

(C)

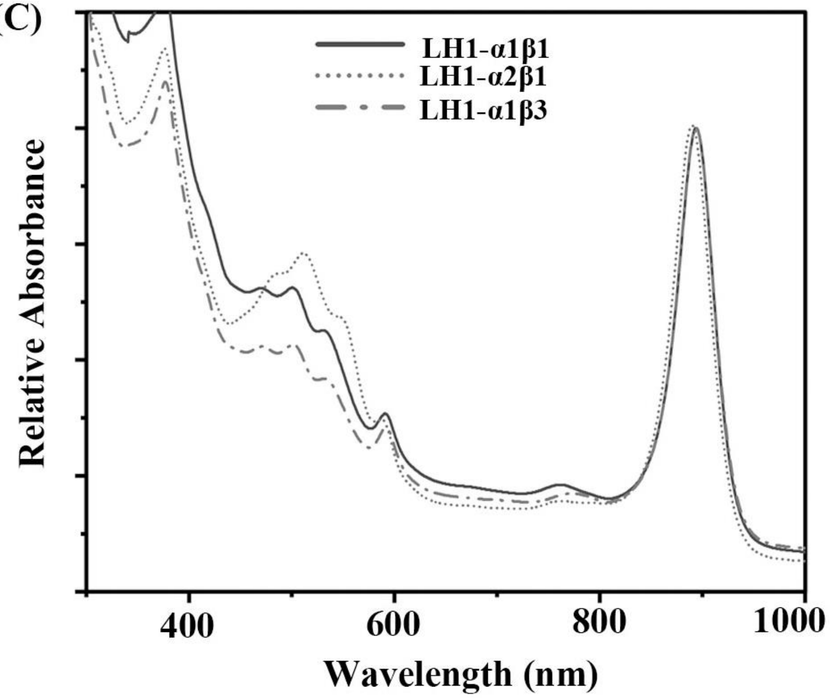

(D)

β1 MANSSMTGLTEQEAQEFHGIFVQSMTAFFGIVVIAHILAWLWRPWL

β3 MADQKSMTGLTEDEAKEFHGIFTQSMTMFFGIVIIAHILAWLWRPWL

Ca

α1 MSPDLWKIWLLIDPRRVLIAVFAFLTILGLAIHMILLSTTEFNWLEDGIPAAKVQQVTPVVPQR

α2 MHKIWQIFDPRRTLVALFGFLFVLGLLIHFILLSSPAFNWLSGS

Fig. S1. Heterologous expression of the *Alc. tepidum* LH1-only complex. Schematic presentations of the arrangement of LH1–RC-encoding genes (*pufBALM*, *puhA*) from *Rsp. rubrum* and the *pufBA* from *Alc. tepidum* subsequent replacement in certain region of *puf* operon. Small arrows represent oligonucleotide primers used for PCR. **(B)** PCR primers used in this study. (**C**) Absorption spectrum of crude ICMs from the constructed strains. (**D**) Sequences of the LH1 polypeptides.

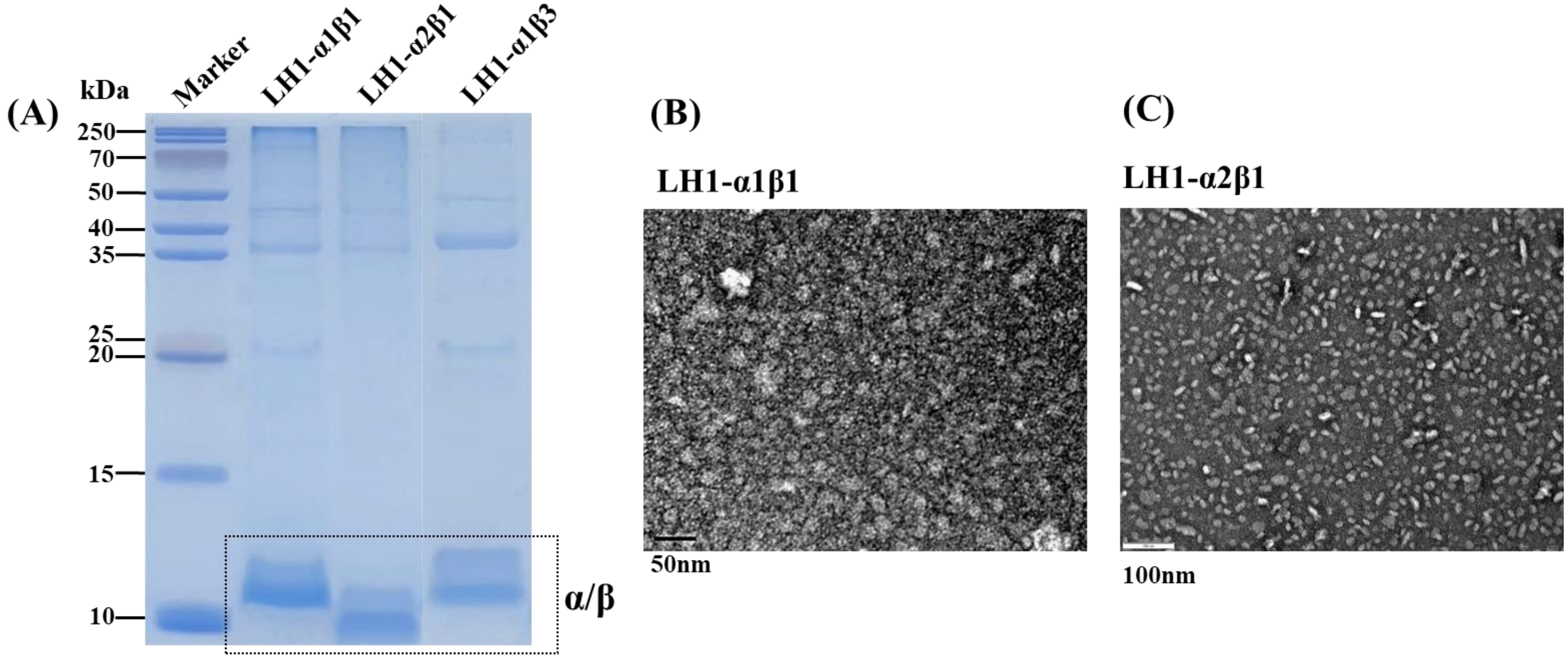

Fig. S2. Characterization of the *Alc. tepidum* LH1-only complexes obtained in this study. (**A**) SDS-PAGE of the purified LH1-only complexes, stained by Coomassie Brilliant Blue. (**B**)(**C**) A representative negatively stained electron micrograph of LH1-α1β1 and LH1-α2β1 with a concentration about ~0.04 mg/mL in 20 mM Tris-HCl (pH 7.5), 0.05% β-DDM.

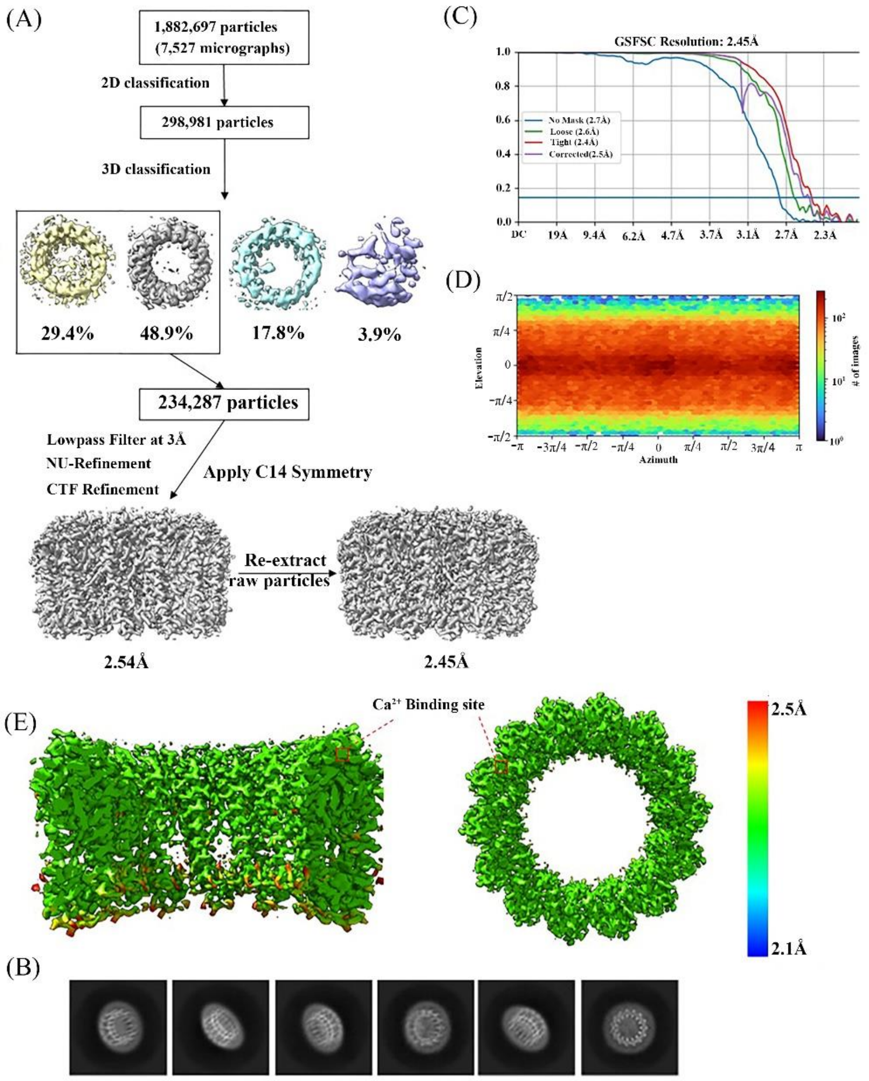

(A)
1,882,697 particles
(7,527 micrographs)
2D classification
298,981 particles
3D classification
29.4%
48.9%
17.8%
3.9%
234,287 particles
Lowpass Filter at 3Å
NU-Refinement
CTF Refinement
Apply C14 Symmetry
Re-extract
raw particles
2.54Å
2.45Å
(C)
GSFSC Resolution: 2.45Å
No Mask (2.7Å)
Loose (2.6Å)
Tight (2.4Å)
Corrected(2.5Å)
1.0
0.8
0.6
0.4
0.2
0.0
DC
19Å
9.4Å
6.2Å
4.7Å
3.7Å
3.1Å
2.7Å
2.3Å
(D)
Elevation
Azimuth
# of images
(E)
Ca²⁺ Binding site
2.5Å
2.1Å
(B)

Fig. S3. Cryo-EM data process of the *Alc. tepidum* LH1-α1β1 complex. (**A**) Image processing flow of 3D classification and reconstruction. Overview of cryo-EM data processing workflow for the *Alc. tepidum* LH1-α1β1 complex dataset. Every major step is shown with the associated number of particles, percentage of particles per class, or estimated resolution. Selected 3D class that went into further processing is marked with rectangle. (**B**) Representative 2D class averages processed from micrograph of LH1-α1β1 complex. (**C**) Gold standard Fourier shell correlation (FSC) plots of the cryo-EM map (unmasked: blue; loose masked: green; tight masked: red; corrected masked: purple) The Fourier shell correlation (FSC) plots of the cryo-EM map (unmasked: blue, loose masked: green, tight masked: red, corrected masked: purple) are superimposed. Global resolution values were calculated according to the gold-standard FSC=0.143. (**D**) Angular distribution of reconstructed particles. (**E**) Local resolution representation of the cryo-EM map of the LH1-α1β1 complex from the side view and top view, respectively.

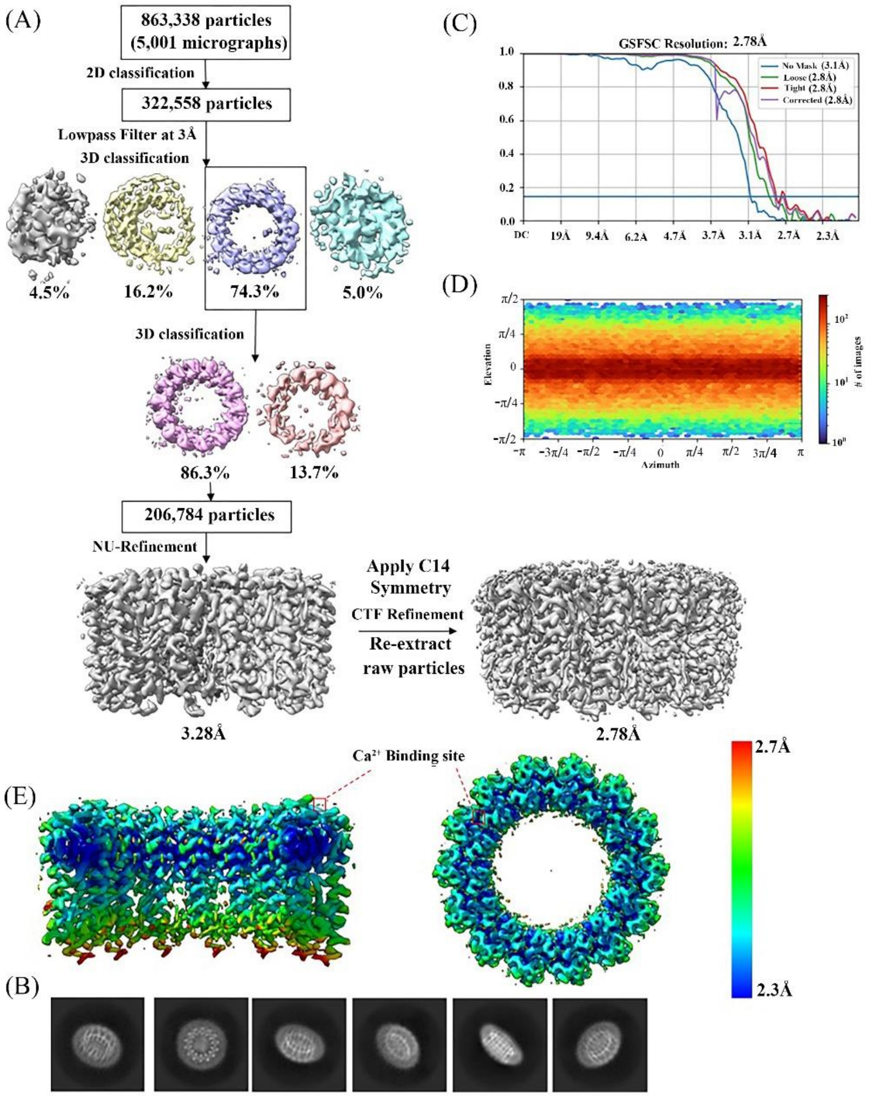
(A)
863,338 particles
(5,001 micrographs)
2D classification
322,558 particles
Lowpass Filter at 3Å
3D classification
4.5%
16.2%
74.3%
5.0%
3D classification
86.3%
13.7%
206,784 particles
NU-Refinement
Apply C14
Symmetry
CTF Refinement
Re-extract
raw particles
3.28Å
2.78Å
(C)
GSFSC Resolution: 2.78Å
No Mask (3.1Å)
Loose (2.8Å)
Tight (2.8Å)
Corrected (2.8Å)
DC
19Å
9.4Å
6.2Å
4.7Å
3.7Å
3.1Å
2.7Å
2.3Å
(D)
Elevation
Azimuth
# of images
(E)
Ca2+ Binding site
2.7Å
2.3Å
(B)

Fig. S4. Cryo-EM data process of the *Alc. tepidum* LH1-α2β1 complex. (**A**) Image processing flow of 3D classification and reconstruction. Overview of cryo-EM data processing workflow for the *Alc. tepidum* LH1-α2β1 complex dataset. Every major step is shown with the associated number of particles, percentage of particles per class, or estimated resolution. Selected 3D class that went into further processing is marked with rectangle. (**B**) Representative 2D class averages processed from micrograph of LH1-α2β1 complex. (**C**) Gold –standard Fourier shell correlation (FSC) plots of the cryo-EM map (unmasked: blue; loose masked: green; tight masked: red; corrected masked: purple) are superimposed. Global resolution values were calculated according to the gold-standard FSC=0.143. (**D**) Angular distribution of reconstructed particles. (**E**) Local resolution representation of the cryo-EM map of the LH1-α2β1 complex from the side view and top view, respectively.

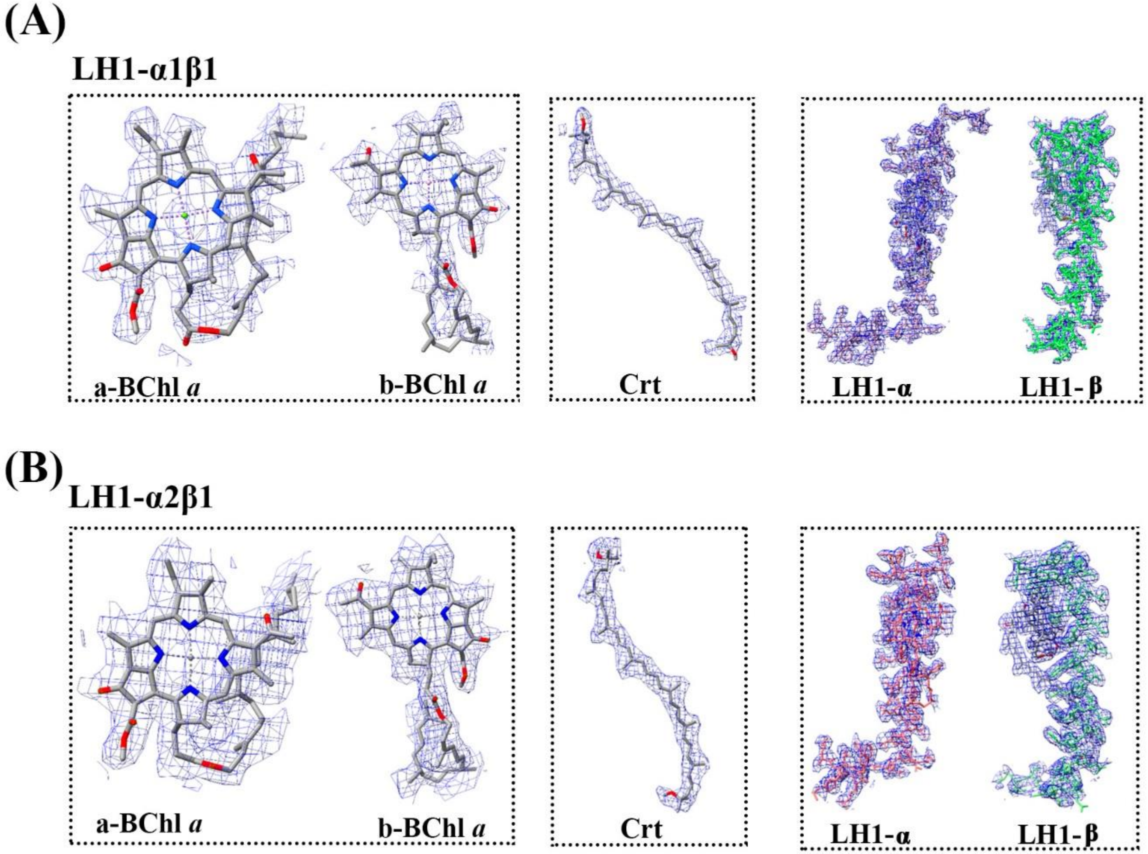

Fig. S5. Cryo-EM densities and structural models of the polypeptides in the *Alc. tepidum* LH1-only complexes. The density maps of LH1-α1β1 (A) and LH1-α2β1 (B) are shown at a contour level of 4.0 σ.

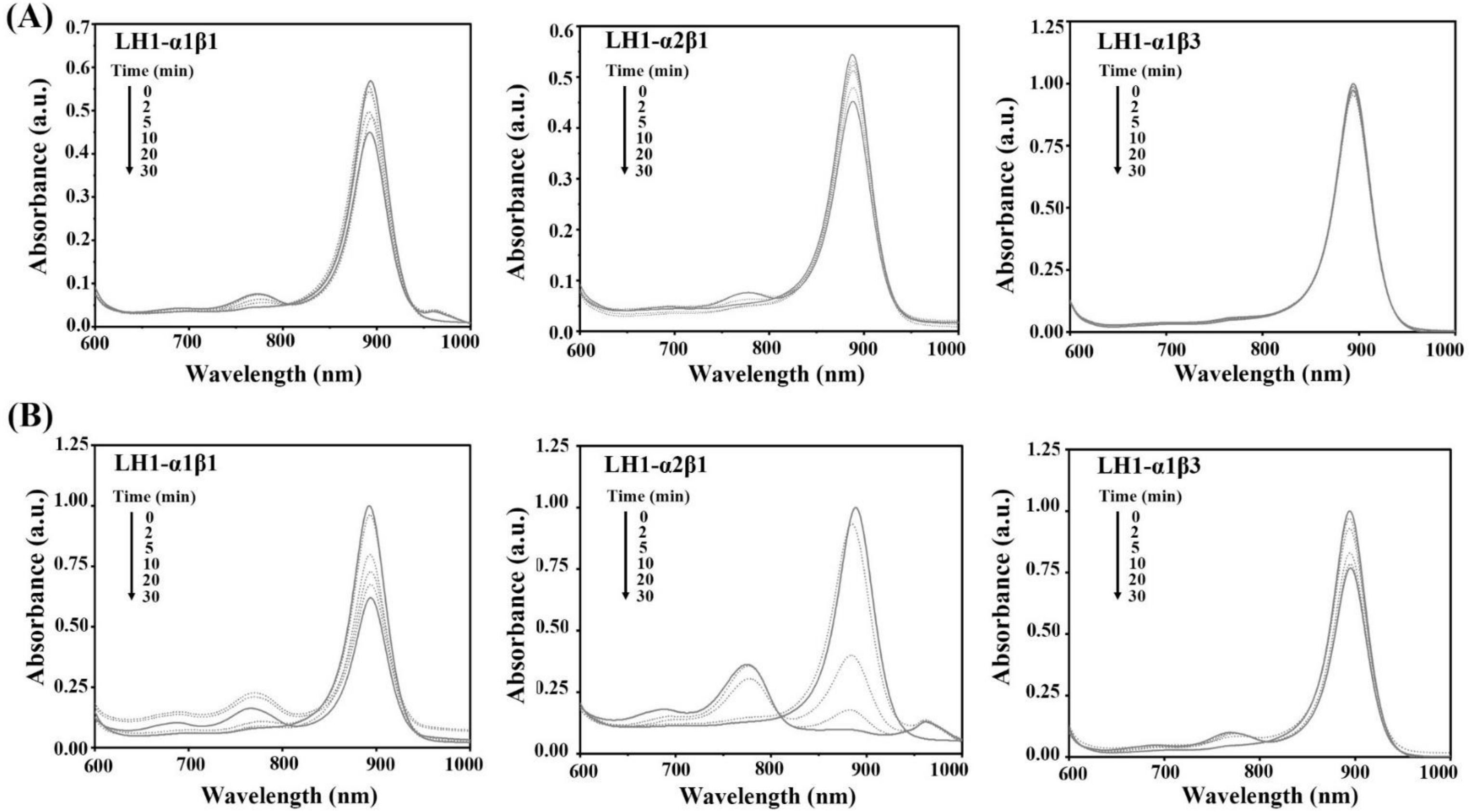

Fig. S6. Changes in the absorption spectra on thermal degradation at 65 °C (A) and 75 °C (B) for the *Alc. tepidum* LH1-only complexes. From left to right: LH1-α1β1, LH1-α2β1 and LH1-α1β3, respectively. The samples were dissolved in 20 mM Tris-HCl (pH 7.5) and 0.03 % (w/v) β-DDM.
